# Progress in Medicine

**Published:** 1898-08-06

**Authors:** 


### Progress in Medicine.

### HEART DISEASE.

**Reduplication of Sounds.**—Sewell[1] comes to the following conclusions in regard to the occurrence of reduplication of the heart sounds. Reduplication of the first sound is a frequent phenomenon, especially in pathological conditions; it may have every degree of completeness, from mere prolongation of the first sound to distinct doubling of the same. The condition is most marked at the end of expiration. These reduplications may be grouped under two heads, as real and simulated. Under the first division fall those reduplications of the first sound due to asynchronism in the contraction of the ventricles. There is also reason for the suspicion that reduplication may have its origin in a double systolic effort of the ventricles. Simulated reduplication of the first sound is probably frequently caused by undue lack of synchronism between the contraction of the ventricle and the tension sound of the corresponding auriculo-ventricular valve. Simulated reduplication may also rise from a post-systolic blow of the pulmonary artery against the chest wall, or to audibility of the auricular contraction.

The prominent factor in causing real reduplication of the first sound is intra- and extra-ventricular blood pressure. As a rule, that ventricle contracts first within which the diastolic blood-pressure is relatively increased. When reduplication of the first sound is perceived in normal subjects it often indicates a lack of cardiac co-ordination, which provokes special symptoms referable to the circulation, *e.g.*, a tendency to faint. True reduplication of the second sound of the heart is produced by asynchronous tension of the two sets of sigmoid valves. The phenomenon is a normal one, the aortic valve sound preceding the pulmonary at the end of inspiration and beginning of expiration. This splitting of the second sound may be wholly absent or only perceptible at about the end of inspiration, or reduplication may be represented by a double sound throughout the respiratory cycle.

The widest reduplication of the second sound occurs when the ventricles are asynchronous in action. When the right ventricle slightly precedes the left the second sound is single, and, conversely, when the second sound is single the contraction of the ventricles is usually asynchronous. When the first sound is single the character of the second sound as regards reduplication depends on the ratio of pulmonic to systemic blood pressure. The greater the difference between these pressures the more marked the reduplication. Therefore, when the lung vessels are congested, or resistance to outflow from them is increased, reduplication of the second sound is diminished or absent. True reduplication of the second sound is heard most plainly at the base of the heart, under the second and third left costal cartilages bordering on the sternum.

The position of the body, and especially respiratory movement, influence reduplication of the second sound in so far as they influence the ratio of pulmonic to systemic arterial pressure. There is reason to believe that the maximum difference between aortic and pulmonary arterial pressure is rhythmically reached at the end of inspiration and beginning of expiration, *i.e.*, in the phase of respiration in which the splitting of the second sound is most marked.

**Tapping the Pericardium.**—Hare[2] remarks that considerable timidity exists in regard to tapping the pericardial sac to relieve a pericardial effusion of dangerous extent. This arises from two reasons—first, by reason of the difficulty in diagnosis between pericardial effusion and cardiac dilatation; and, second, because the fear exists that the heart may be wounded.

From an examination of cases recorded in medical literature Hare comes to the conclusion that wounds of the heart are not necessarily followed by serious results. Death, in case of heart wounds, may be due (1) to hæmorrhage, which results in loss of blood or distension of the pericardium with blood causing paralysis of the heart's action; (2) to hæmorrhage from the heart muscle itself, which is unusually slight; (3) to wounding of the coronary artery; or (4) to injury to a spot above the lower limit of the upper third of the ventricular septum which brings the heart to a standstill. The lethal result of a heart wound depends then largely on the rapidity with which a hæmostatic clot is formed in the opening, whilst injury to the heart muscle itself is rarely the cause of death.

In the case of paracentesis pericardii danger from puncturing the heart is probably largely overdrawn, for the opening made by a small aspirating cannula is never large enough to produce hæmorrhage from the ventricle.

**Mitral Stenosis.**—Sansom[3] considers that the most frequent cause of mitral stenosis is a slowly developing endocarditis of the rheumatic form, whilst a far less frequent cause, and one operating for the most part in advanced life, is a general widely spread, slowly developing fibrosis. He finds that the hypothesis of any tuberculous causation is not supported by anything like sufficient evidence. In some cases of mitral stenosis of the funnel form a systolic murmur is heard in the neighbourhood of the apex. This murmur is probably often due to regurgitation through the tricuspid orifice. If, then, mitral regurgitation does not occur in many cases of mitral stenosis, the question occurs how is such regurgitation prevented? The answer is to be found in the fact that the characteristic modification of the left auricle in this affection is hypertrophy, dilatation occurring only with failure of compensation. This hypertrophied auricle goes on contracting until the contracting ventricle begins to empty its blood into the aorta. There are other factors as well tending to prevent regurgitation into the auricle, *i.e.*, the diminution in the area of the auriculo-ventricular ring, and the shortening and approximation of the papillary muscles. Again, the auricular cavity being small (its muscular walls by their contraction having nearly emptied it) in comparison with the ventricular cavity, it is possible that the auricle as a contracting sphere may be master of the situation, and force the remnant of its blood into the ventricle in spite of the muscle of the latter being already in contraction. This hypertrophy of the left auricle in cases of mitral stenosis offers an explanation of a fact long ago observed by Dickinson—that the murmur in some cases of mitral stenosis occurs not before, but simultaneously with the elevation of the heart's apex, as observed by the finger. Dickinson's explanation of this was that the murmur was not auricular and pre-systolic, but systolic and of ventricular causation. Potain and Sansom have shown that the muscular contraction of the hypertrophied auricle in some cases of mitral stenosis is so great as to lift the apex of the ventricle. In such cases the murmur is heard synchronously with the apex beat, but this apex beat is due to distention of the ventricle and not to the systole of the ventricle, which follows later. Dickinson's observations, then, were accurate, though the inference was unsound.

The prognosis in cases of mitral stenosis is grave. Samways finds the average age at death is 38⅓ years; the less pronounced forms of constriction of the orifice 43 years; the more extreme stenosis 33 years. Life then is rarely prolonged beyond 40 years in mitral stenosis of rheumatic origin. In the form of disease associated with chronic fibrosis and granular kidney the age at death is much more advanced.

[1] Sewell, Amer. Jour. Med. Sci., July, 1893. [2] Hare, Therap. Gaz., Feb. 15, 1898. [3] Sansom, Brit. Med. Jour., June 25, 1898.

